# *Mesorhizobium ciceri* as biological tool for improving physiological, biochemical and antioxidant state of *Cicer aritienum* (L.) under fungicide stress

**DOI:** 10.1038/s41598-021-89103-9

**Published:** 2021-05-06

**Authors:** Mohammad Shahid, Mohammad Saghir Khan, Asad Syed, Najat Marraiki, Abdallah M. Elgorban

**Affiliations:** 1 Department of Agricultural Microbiology, Faculty of Agricultural Sciences, Aligarh Muslim University, Aligarh, 202002 Uttar Pradesh India; 2 Department of Botany and Microbiology, College of Science, King Saud University, P.O. Box 2455, Riyadh, 11451 Saudi Arabia; 3 Center of Excellence in Biotechnology Research, King Saud University, Riyadh, Saudi Arabia

**Keywords:** Microbiology, Environmental sciences

## Abstract

Fungicides among agrochemicals are consistently used in high throughput agricultural practices to protect plants from damaging impact of phytopathogens and hence to optimize crop production. However, the negative impact of fungicides on composition and functions of soil microbiota, plants and via food chain, on human health is a matter of grave concern. Considering such agrochemical threats, the present study was undertaken to know that how fungicide-tolerant symbiotic bacterium, *Mesorhizobium ciceri* affects the *Cicer arietinum* crop while growing in kitazin (KITZ) stressed soils under greenhouse conditions. Both in vitro and soil systems, KITZ imparted deleterious impacts on *C. arietinum* as a function of dose. The three-time more of normal rate of KITZ dose detrimentally but maximally reduced the germination efficiency, vigor index, dry matter production, symbiotic features, leaf pigments and seed attributes of *C. arietinum*. KITZ-induced morphological alterations in root tips, oxidative damage and cell death in root cells of *C. arietinum* were visible under scanning electron microscope (SEM). *M. ciceri* tolerated up to 2400 µg mL^−1^ of KITZ, synthesized considerable amounts of bioactive molecules including indole-3-acetic-acid (IAA), 1-aminocyclopropane 1-carboxylate (ACC) deaminase, siderophores, exopolysaccharides (EPS), hydrogen cyanide, ammonia, and solubilised inorganic phosphate even in fungicide-stressed media. Following application to soil, *M. ciceri* improved performance of *C. arietinum* and enhanced dry biomass production, yield, symbiosis and leaf pigments even in a fungicide-polluted environment. At 96 µg KITZ kg^−1^ soil, *M. ciceri* maximally and significantly (*p* ≤ 0.05) augmented the length of plants by 41%, total dry matter by 18%, carotenoid content by 9%, LHb content by 21%, root N by 9%, shoot P by 11% and pod yield by 15% over control plants. Additionally, the nodule bacterium *M. ciceri* efficiently colonized the plant rhizosphere/rhizoplane and considerably decreased the levels of stressor molecules (proline and malondialdehyde) and antioxidant defence enzymes viz. ascorbate peroxidise (APX), guaiacol peroxidise (GPX), catalase (CAT) and peroxidises (POD) of *C. arietinum* plants when inoculated in soil. The symbiotic strain effectively colonized the plant rhizosphere/rhizoplane. Conclusively, the ability to endure higher fungicide concentrations, capacity to secrete plant growth modulators even under fungicide pressure, and inherent features to lower the level of proline and plant defence enzymes makes this *M. ciceri* as a superb choice for augmenting the safe production of *C. arietinum* even under fungicide-contaminated soils.

## Introduction

*Cicer arietinum* L. (chickpea) crops often suffers from the attack of phytopathogens, which damage the crop and consequently limits its yield. Fungicides are commonly used to enhance productivity by preventing phytopathogen-related damage^1^. However, massive and injudicious use of such chemicals can upset soil fertility and inhibit microbial communities^2^ and enzymatic activities^3^. Apart from the cytotoxic and genotoxic effect of fungicides on soil microbiota, uptake and translocation of fungicides by different plant organs may severely damage important metabolic activities leading subsequently to death of plants^4^. Exceptionally high concentrations of pesticides disrupt: (i) cellular organelles and membrane permeability^5^; (ii) respiratory processes and carbohydrate metabolism^6^; (iii) physiologically active enzymes^7^ and proteins^8^; (iv) photosystems by blocking the effective quantum yield of PSII (ΦPSII) and quantum efficiency of PSII (Fv/Fm)^9^; and (v) cause oxidative damage^10^ and alter the genetic makeup^11^. Zablotowicz and Reddy^12^ observed that the pesticide glyphosate considerably decreased nitrogenase activity of rhizobia. As a consequence, symbiotic events leading to nodule formation and root morphogenesis of plants were drastically diminished^13^. In another study, the fungicide pyrimorph was found to strongly inhibit the electron transport (ET) reactions of chloroplasts and adversely affected the physiology of whole plants^14^.

To overcome these problems, certain physico-chemical approaches have been used to limit or destroy the toxic effects of pesticides. However, these methods are expensive, and the remediation process often remains incomplete due to the transformation of the parent compound to metabolites which are sometimes more persistent and more toxic for neighboring non-target organisms than were the parent compounds. As a consequence, physico-chemical methods of pesticide removal have not been widely accepted in crop cultivation practices. The search for alternative strategy for pesticide degradation/detoxification therefore, becomes eminent. In this regard, the bioremediation offers some solutions to the pesticide detoxification problems. The technique, which relies on the use of soil microbiota, often referred to as ‘microbial remediation’ is gaining impetus to convert contaminants to simpler and harmless forms and hence, to mitigate the pesticide pollution.

To this end, scientists have identified pesticide degrading/detoxifying microbes endowed with potential plant growth promoting activities. Chief among them belongs to genera *Ensifer*^15^, *Bradyrhizobium*^16^, *Rhizobium*^17^, *Alcaligenes*^18^, *Actinobacteria*^19^ and *Bacillus*^20^. Apart from degradation of toxic pollutants, plant growth promoting rhizobacteria (PGPR) have the ability to synthesize growth regulating substances. By expressing multifarious physiological activities, PGPR promotes the overall growth and yield of legumes^21^ raised in soil contaminated during cultivation with pesticides. For instance, inoculation of *Azotobacter*^22^ and *Bacillus*^23^ had positive effects on pulses where they supplied N and growth stimulating substances including phytohormones, siderophores and EPS, and solubilized soil P. Some fungicide-tolerant and N_2_-fixing bacterial strains such as *Rhizobium* and *Azotobacter* and Gram-positive *Bacillus* sp. detoxified pesticides and enhanced legume production under adverse conditions^24^. Likewise, pesticide-tolerant free-living PGPR such as *Bacillus*^25^, *Azotobacter*^26^ and *Stenotrophomonas*^27^ circumvented the toxicity of pesticides and concurrently improved growth of legumes.

Given the nutritive importance of *C. arietinum* in the global dietary systems, the negative impact of fungicides on legume production, the lack of adequate information on fungicidal response to *C. arietinum* and the inherent bioremediation potential of PGPR, this study was formulated to explore the following: (i) the fungicidal toxicity to *C*. *arietinum* both under in vitro bioassays and in pot-house conditions (ii) the kitazin-induced distortion, oxidative damage and cell death in *C. arietinum* root cells (iii) to identify fungicide tolerant symbiotic bacterium from chickpea nodules (iv) to determine the production of bioactive molecules under fungicide stress (v) to evaluate the effects of *M. ciceri* on physiological and biochemical attributes of *C*. *arietinum* (vii) to determine the impact of stressor molecules on antioxidant enzymes of *C. arietinum* foliage detached from fungicide-treated and *Mesorhizobium*-inoculated chickpea and (viii) to evaluate the rhizosphere/rhizoplane colonization efficiency/competence ofnodule bacterium .

## Materials and methods

### Toxicity assessment of Kitazin to *Cicer arietinum* under in-vitro conditions

#### Seed germination, vigor index and plant length

Healthy and vigorous seeds of *C. arietinum* L. (desi chickpea) (cv. avarodhi) were soaked in water to imbibe for 24 h. Seeds were disinfected with 3% sodium hypochlorite (NaOCl) solution and carefully rinsed with sterile water. Soft agar (0.7%) plates supplemented with different concentrations of KITZ (for detail, see Supplementary Table S1) were poured. Soft agar plates without fungicide treatment were served as a control for comparison. Seeds were placed on the soft agar plates and kept at room temperature (28 ± 2 °C). After 6–7 days, germination percentage, vigor index, radicle and plumule lengths of plantlets were recorded.

#### Percent phytotoxicity, tolerance index (TI) and root-shoot length ratio

The phytotoxicity (%), tolerance index and root: shoot of length was recorded in fungicide treated/untreated *C. arietinum* seedling. (See the electronic supplementary information for details).

### Micro-morphological alteration, oxidative damage and cytotoxicity in *C. arietinum* root

#### Alteration in root tip morphology analyzed by scanning electron microscope (SEM)

The toxic and damaging/destructive impact of fungicide on root morphology of *C. arietinum* plants grown on soft agar plates treated with 1000 μg KITZ mL^−1^ was monitored under scanning electron microscopy (JSM 6510 LV, JEOL, Japan). For this, disinfected seeds were germinated on 0.7% soft agar for seven days at room temperature. Briefly, root samples were washed three times with phosphate buffer and then fixed for 12 h in 2% (v/v) glutaraldehyde prepared in 0.1 M phosphate buffer (pH 7.0), washed three times with the same buffer and dehydrated in a graded series of ethanol (30%, 50%, 70%, 90% and 100%) at 4 °C. Samples were dried to critical point dryer (CPD), fixed in gold stubs and examined under SEM to check the fungicide induced distortion/damage in root tips, if any.

#### Oxidative damage and cell death by confocal laser scanning microscopic (CLSM) analysis

Fungicide induced cellular/oxidative damages in root tissues of *C. arietinum* were viewed under CLSM. For the assay, sterilized seeds of *C. arietinum* were allowed to germinate on 0.7% soft agar plates amended with 0 (untreated control), 500 μg mL^−1^ (1 ×), 1000 μg mL^−1^ (2 ×) and 1500 μg mL^−1^ (3 ×) concentrations of KITZ at room temperature. After 7–8 days, the roots were carefully detached from soft agar and washed with buffer to remove the agar. After successive washing, root samples were tagged with a mixture of two fluorescent tags (i) acridine orange (10 μg mL^−1^) and (ii) propidium iodide (25 μM) and mounted on a glass slide. The stained roots were observed for increasing dead tissues with progressively increasing fungicide concentrations under LSM-780 Confocal Microscope (Zeiss, Germany).

In order to differentiate between metabolically active and inactive cells, loss of cell viability in root tissues of *C. arietinum*, the cellular membrane was used as a toxicity indicator. A well-adapted Evans blue staining procedure was followed as described previously by Baker and Mock^28^. *C. arietinum* roots grown in the presence of three concentrations of KITZ were allowed to take up the 0.25% w/v solution of Evans blue stain for at least 15 min. and successively washed three times with double distilled water (DDW). The emission of fluorescence was examined under Confocal Microscope to assess the cell death in root tissues.

### Isolation of root nodule bacteria and Kitazin tolerance

Fresh, un-damaged and healthy nodules, detached from chickpea plants were surface sterilized by dipping the nodules in 4% NaOCl for 2 min., washed three times with sterile double distilled water (DDW) and crushed gently. A-100 μL freshly extracted nodule suspensions was streaked on Yeast extract mannitol agar (YEMA) plates and incubated at 28 ± 2 °C for 3–5 days. A total of 20 *Mesorhizobium* isolates were recovered and identified to genus level by morphological and biochemical tests^29^. Plant infection technique was performed to ascertain the host specificity of selected nodule strains^30^. Mesorhizobial isolates were exposed to varying concentrations of kitazin using minimal salt agar (MSA) in order to select fungicide tolerant *Mesorhizobium* isolates. Colonies grown on YEMA plates and efficiently surviving at the highest concentration of KITZ were chosen and referred to as fungicide tolerant mesorhizobial strains. Of the total 20 *Mesorhizobium* isolates, BRM5 expressing maximum tolerance to pesticides was selected for further studies.

### Molecular identification of BRM5 isolate

For the identification of isolate to species level, 16S rRNA partial gene sequencing was performed using universal primers 785F (5′-GGATTAGATACCCTGGTA-3′ and 907R (5′-CCGTCA ATTCMTTTRAG TTT-3′)^31^. The nucleotide sequence data obtained from Macrogen was deposited in the GenBank sequence database. The BLASTn program available online was employed to find similar sequences with known taxonomic information accessible from the databank accessible at the NCBI website (http://www.ncbi.nlm.nih.gov/BLAST)^32^ (See the supplementary method section for detailed methods of DNA isolation, PCR and sequencing).

### Production of PGP active biomolecules under fungicidal pressure

#### Indole acetic acid (IAA), siderophore production and 1-amino cyclopropane 1-carboxylate (ACC) deaminase activity

The IAA produced by strain BRM5 was quantitatively assessed by modified method of Brick et al*.*^32^. For the assay, a-100 mL LB broth containing fixed concentration of tryptophan (100 mg mL^−1^) was treated with 0, 600 (1 ×), 1200 (2 ×) and 1800 (3 ×) µg mL^−1^ KITZ. The fungicide containing LB was inoculated with 100 μ *M*. *ciceri* BRM5 (10^8^ cells m^−1^) and incubated at 28 ± 2 °C for four days with shaking at 120 r/min. Following complete incubation, culture (5 mL) was centrifuged (8000 r/min) for 10 min. and two mL supernatant was added with 100 μL orthophosphoric acid and four mL Salkowsky reagent (2% 0.5 M FeCl_3_ prepared in 35% per-chloric acid) and incubated for one hour at 28 ± 2 °C in darkness for color development. The absorbance of pink colour appearing during reaction was measured at 530 nm and the IAA was quantified by calibrating against pure IAA, as a standard.

The rhizobial isolate was spot inoculated on kitazin supplemented chrome azurol S (CAS) agar plates followed by incubation at 28 ± 2 °C for detection of orange coloured halo around the rhizobial colonies. The siderophore was assessed further quantitatively by growing the rhizobial strain in fungicide amended iron (Fe) free succinate liquid medium as suggested by Barbhaiya and Rao^33^. The amount of siderophore was estimated according to universal chrome azurol liquid assay^34^ and siderophore units were calculated as-:

$$\% \, \;{\text{Siderophore }}\;{\text{unit }} = \frac{{{\lambda }\;{\text{of }}\;{\text{reference }}\left( {{\text{Ar}}} \right) - {\lambda }\;{\text{of }}\;{\text{test }}\left( {{\text{As}}} \right)}}{{{\lambda }\;{\text{of }}\;{\text{reference }}\left( {{\text{Ar}}} \right)}} \times 100$$

where, Ar = Absorbance of un-inoculated media and CAS solution, As = Absorbance of test sample i.e. culture supernatant and CAS solution.

The plant growth modulating enzyme ACC deaminase (EC 4.1.99.4) secreted by *M*. *ciceri* BRM5 was qualitatively detected by spot inoculation method using Dworkin and Foster (DF) salts minimal medium^35^ containing 3 mM ACC as N source. Plates containing DF medium without ACC and with (NH_4_)_2_SO_4_ (0.2% w/v) served as negative and positive control, respectively. Plates maintained at 28 ± 2 °C for 72 h were examined each day for bacterial growth. *Mesorhizobium* LMS-1 containing pRKACC plasmid^36^ was used as a positive control. The ACC deaminase secreted by bacterial strain grown at 1 ×, 2 × and 3 × of fungicides was extracted as suggested by Honma and Shimomura^37^ and Penrose and Glick^38^. The quantity of α-ketobutyrate generated due to ACC deaminase activity was measured spectrophotometrically against a standard curve of α-ketobutyrate. The activity of ACC deaminase was expressed as the quantity of α-ketobutyrate released/mg of protein/h. All individual experiments were conducted three times on different intervals.

#### EPS, HCN and ammonia production

The exopolysaccharide (EPS) released by *M. ciceri* BRM5 was extracted by the method of Mody et al*.*^39^. For this, bacterial cultures were grown in nutrient broth supplemented with 5% sucrose and treated with 0, 1 ×, 2 × and 3 × doses of KITZ and incubated for 5 days at 28 ± 2 °C at 120 rpm. Culture broth was centrifuged (8000 rpm min^−1^) for 20 min and EPS was extracted by mixing chilled acetone (CH_3_COCH_3_) and supernatant in a ratio of 3:1. The precipitated EPS so obtained was washed three times alternately with distilled water and acetone and transferred to filter paper and weighed after overnight drying at room temperature.

The production of cyanogenic compound (HCN) and ammonia were assayed using the methods of Bakker and Shipper^40^ and Dye^41^, respectively (See supplementary methods).

### Crop-based experiments

#### Planting, fungicide treatment and application of mesorhizobium

Chickpea seeds were disinfected/sterilized with NaOCl (2%), washed, cleaned and desiccated at room temperature. Commercial grade fungicide kitazin (Table S1) [recommended dose: 1 × (96 µg Kg^−1^), 2 × (192 µg Kg^−1^) and 3 × (288 µg Kg^−1^)] of soil were applied to moist experimental soils one day before sowing of seeds. . The soils were filled in 20 × 24 cm clay pots containing approximately 5 kg soil/ pot. Seeds were then coated/bacterized with freshly prepared inoculum of *M. ciceri* after dipping the seeds in liquid culture medium for 2 h using 10% gum arabic as a sticker to achieve 1 × 10^8^ cells seed^−1^ which was confirmed by viable cell count. The un-inoculated sterilized seeds submerged in sterile water only were taken as control. Non-bacterized and bio primed seeds (n = 10) were sown in respective earthen pots containing 5 kg of conventional soils. Two controls were run in parallel; one was un-inoculated and untreated control (without bacteria and without fungicides) and another was inoculated (only bacteria but no fungicides). Each test concentration was replicated three times and pots were arranged in a completely randomized block design. After germination, seedlings were thinned and two uniform healthy seedlings of *C. arietinum* were maintained in each pot, 15 days after emergence (DAE). Pots were watered regularly and were kept in an open field condition (9 h photoperiod/15 h dark cycle. At the time of chickpea sowing, the temperature during day and night was: average day/night in November (28.3/12.9 °C), December (22.5/9.5 °C), January (20.6/7.4 °C), and February (23.6/9.5 °C). The crop experiments were performed regularly for two succeeding years to achieve the consistency in results.

#### Germination efficiency, plant height, dry biomass and photosynthetic pigment in the presence of *M. ciceri* and KITZ

The KITZ treated and bacterized *C. arietinum* plants were removed at 80 and 120 days after sowing (DAS) and germination efficiency, root and shoot length, weight and dry biomass was measured. For dry biomass, plants were dried in oven (Yorco, York Scientific Industries, Pvt. Ltd. India) at 80 °C for 2 days and calculated. Leaf photosynthetic molecules (chlorophyll and carotenoid) accumulated in fungicide treated/untreated and bacterized *C. arietinum* foliage was estimated following the methods of Arnon^42^ and Kirk and Allen^43^, respectively (See supplementary methods in electronic supporting information).

#### Symbiosis, nutrient uptake and seed attributes

Symbiotic features of *C. arietinum* were assayed by carefully removing the nodules from root systems. Nodules were counted and oven dried (80 °C) in a ventilated oven for 48 h. After drying, nodule dry biomass (mg plant^-1^) was weighed and average was calculated. Furthermore, leghaemoglobin (LHb) content was quantitatively assayed as previously described by Shahid and Khan^44^ (See electronic supporting information).

The nutritional content (nitrogen and phosphorous) in fungicide treated and bacterized *C. arietinum* plants was estimated at harvest (120 DAS) as previously described by Jackson^45^ and Lindner^46^, respectively. *C. arietinum* plants grown in treated/untreated and bio-inoculated sandy clay loam soils were finally harvested at 120 DAS and seed yield (SY) was recorded. Grain protein was extracted and estimated following the method of Lowry^47^. (See supplementary methods).

### Assessment of oxidative stress in bio inoculated and fungicide treated *C. arietinum* plants

#### Estimation of proline

The free prolinecontent in various organs of *C. arietinum* cultivated with/without fungicide was assayed as described earlier by Bates et al*.*^48^ (See supplementary methods).

#### Estimation of MDA content (lipid peroxidation)

Lipid peroxidation in leaf tissues of *C. arietinum* was measured as malondialdehyde (MDA). Absorbance of abduct (MDA-TBA2) after reaction of thiobarbituric acid (TBA) and MDA was measured spectrophotometrically^49^. For the assay, fresh foliage (500 mg) was homogenized with 10 mL tri-chloroacetic acid (TCA; 5% w/v) on an ice bath followed by centrifugation (12,000×*g*) at 4 °C for 20 min. Equal volumes of resulting supernatant and thiobarbituric acid (TBA; 0.67% w/v) (HiMedia Pvt. Ltd. India) were mixed in a glass tubes followed by heating at 100 °C in a water bath for 30 min, and then kept on an ice-bath to terminate the reaction. After centrifugation (10,000×*g*) at 4 °C for 10 min, the optical density of supernatant was recorded at three wavelengths (λ) of 450, 532, and 600. The MDA levels were calculated using the following equation and molar extinction coefficient of 155 mM^−1^ cm^−1^.

$${\text{MDA}}\;{\text{ level }}\left( {{\upmu }\;{\text{mol}}/{\text{L}}} \right) \, = { 6}.{45} \times \, \left( {\lambda_{{{532}}} {-} \, \lambda_{{{6}00}} } \right) \, {-} \, 0.{56 } \times \, \lambda_{{{45}0}}$$

### Extraction and determination of antioxidant enzymes

For antioxidant enzyme activity, foliage was crushed in 4 mL of enzyme extraction buffer [(50 mM phosphate buffer (pH 7.8)] containing 1 mM ethylenediaminetetraacetic acid (EDTA) and 2% (w/v) polyvinylpyrrolidone (PVP). For guaiacol peroxidase (GPX; E.C. 1.11.1.7), foliage tissues (100 mg) were homogenized in tris-buffer and the homogenate was centrifuged at 12,000 rpm for 20 min. at 4 °C. Increase in absorbance at 470 nm due to formation of tetra guaiacol (ε = 26.6 mM^−1^ cm^−1^) is expressed as µ mol mg protein^−1^ min^−1^. The reaction mixture (3 mL) consisted of 100 mM phosphate buffer (pH 7.0), 0.1 mM EDTA and 20 mM H_2_O_2_. The reaction was initiated by adding 100 µL of enzyme extract. The peroxidases (POD; E.C. 1.11.1.7) and ascorbate peroxidase (APX; E.C. 1.11.1.11) activities were determined following the modified methods of Leonards et al*.*^50^ and Hammerschmidt et al.^51^, respectively. All enzyme assays were performed three times with three replicates of each assay.

### Rhizosphere and rhizoplane colonization by *M. ciceri* under stress

The colonization of root surface by *M. ciceri* was determined in the presence/absence of fungicide. For the examination, roots were rinsed with DDW and PBS. The scanning electron microscopy was performed following the method of Shahid et al*.*^52^. Furthermore, the colonization of roots in term of CFU g^−1^ of root material was determined at 40 and 80 DAS after exposure to different concentrations of fungicides.

### Statistical analyses

The data were statistically analyzed using Sigma Plot 12.0 and Minitab17 software. Completely randomized design (CRD) for pot experiments was followed with at least three pots per individual test concentration. Crop experiments were conducted for two consecutive years to confirm the reproducibility of data. The data recorded in each year were pooled and analyzed. The mean of the data within a single column was calculated and compared with control treatments. The data obtained for in vitro study (non-plant) were analyzed statistically by Duncan’s multiple range (DMRT) test. The data represented either in figures or tables is the mean ± standard deviation (S.D.) of at least three replicates (n = 3). Different alphabets in graphs and tables show a significant difference among the treatments at a confidence level of *p* ≤ 0.05. The least significant difference (LSD) among treatment means was calculated by two-way analysis of variance (ANOVA) at *p* ≤ 0.05.

## Results and discussion

### Toxic impact of fungicide on *C. arietinum* under in-vitro conditions

#### Germination percentage, vigor index, length, phytotoxicity%, tolerance index (TI) and root-shoot length ratio

**Figure 1:**
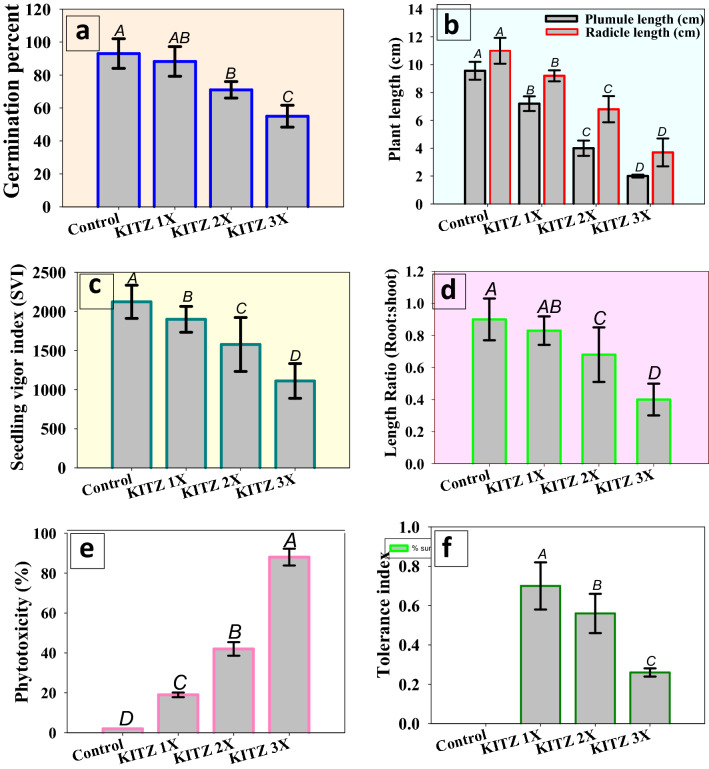
Effect of kitazin on germination percent (**a**), plant length (**b**) vigor index (**c**) root-shoot length ratio (**d**) percent phytotoxicity (**e**) and tolerance index (**f**) of *Cicer arietinum* seedlings geminated on 0.7% soft agar plates treated with three concentrations of KITZ under in vitro condition. Each bar represents the mean ± S.D (*n* = 3) of three replicates where each replicate constituted three plants/pot. Mean values followed by different letters are significantly different at *p* ≤ 0.05 according to Duncan’s multiple range (DMRT) test whereas error bars represent standard deviation (S.D).

The impact of varying dose of KITZ on germination efficiency and seedling attributes of *C*. *arietinum* seed developed on fungicide-amended agar plates though differed but in general it was negative (Fig. 1). The 3 × concentration showed pronounced toxicity and significantly (*p* ≤ 0.05) reduced the germination %, vigor index (SVI), and radicle (RL) and plumule (PL) length by 40%, 47%, 66% and 79% compared to control, respectively (Fig. 1a–c). The reduction in germination efficiency and vigor index may possibly due to the distressed germination metabolism caused by the fungicide. Detrimental impact of pesticides on the germination ability of other legumes have been reported previously . In this regard, lethal effect of fungicides on seedling germination and biological attributes of *P. sativum* under in vitro conditions has been reported^53^. A—19%, 42% and 88% phytotoxicity was recorded for *C*. *arietinum* when grown with 96, 192 and 288 μg KITZ kg^−1^, respectively, compared to control (Fig. 1e). The higher KITZ concentration (3 ×) maximally affected the root-shoot length ratio and reduced it from 0.9 to 0.4 (55% reduction over control) (Fig. 1d). The tolerance index (TI) for *C. arietinum* decreased with increasing KITZ dose and confirmed a negative correlation between fungicide and TI. The TI of *C.* *arietinum* was recorded lowest as 26% at 3 × rate of KITZ over untreated control (Fig. 1f). These results indicate that lower fungicide concentrations resulted in maximum TI, whereas the 3 × dose exhibited the minimum TI in *C. arietinum*. Similarly, root-shoot length ratio and tolerance index of chickpea were negatively influenced by the higher concentrations of two neonicitnoid group of pesticides^54^.

### Micro-morphological root tip distortion, oxidative stress and cell death

**Figure 2:**
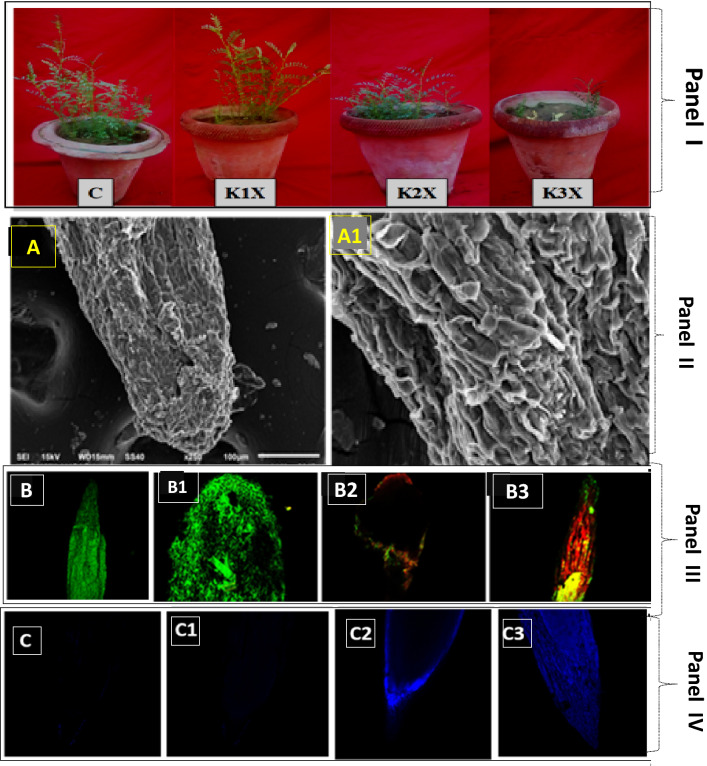
Effect of KITZ on *C. arietinum* plants grown with 96 (KITZ 1 ×), 192 (KITZ 2 ×) and 288 µg KITZ kg^−1^ (KITZ 3 ×) soil (**I**). Scanning electron microscopic (SEM) images of *C. arietinum* roots demonstrating distortion/damage induced by KITZ exposure: (**A**) represents the root tip and root surface of untreated/control. Whereas, (**A1**) represent the distorted/ruptured root tips and root tip surfaces treated with KITZ (**II**). The Z-stack images of PI/AO stained *C. arietinum* roots using CLSM. Images reveal an increase in red/orange fluorescence as concentrations of KITZ increase. Untreated control root showing no red color (**B**), while roots treated with various doses of KITZ (**B1–B3**) (**III**). The Z-stack image of chickpea using CLSM representing the cytotoxicity (Evans blue dye exclusion) assay in root tissues induced by fungicide. Figures show uptake of Evans blue dye by root cells; untreated control root showing no blue color (**C**), while (**C1**–**C3**) represents the roots treated with various doses of KITZ (**IV**).

The SEM images revealed the inhibitory effects of fungicide to the radicle regions of root tips and their surfaces, which were visible as cracks/fractures, breakages, crumbling and spears relative to an indistinct, smooth/even and unbroken shape (Fig. 2II B,B1) as shown in the control root tip and surface (Fig. 2II A,A1). The alterations in root tip morphology further validates the fungicide toxicity which, in turn, might have reduced the uptake of water and nutrients from soil causing altered roots as well as reduced plant growth (Fig. 2I). Similar damage to the micro-morphological structure of roots tips due to the toxicity of pesticides and other toxic pollutants have been reported earlier^55–57^.

Fungicide-induced oxidative stress in root membranes was also visualized. AO/PI stained and fungicide-treated *C.* *arietinum* roots were observed using CLSM. A concentration-dependent increase in dead/injured cells was seen as red/orange color occurred in roots exposed to 3 × of KITZ (Fig. 2III B1–B3).

Untreated root tissues exhibited maximum intensity of green fluorescence resulting from AO reaction representing little or no damage (Fig. 2III B). This is an indication that pesticide exposure was arbitrated by ROS-mediated damage to membrane lipids which therefore increased fluorescence of DNA-bound propidium iodide in membranes. Cortés-Eslava et al*.*^58^, using CLSM, reported similar oxidative stress, oxidative damage and apoptosis in two model plants grown in insecticide- stressed conditions. The loss/damage of plasma membrane in fungicide-treated root tissue was obvious when *C. arietinum* roots were stained with Evans blue dye. The uptake of dye by root tissues increased three- to four-fold with increasing KITZ concentrations (Fig. 2IV C1–C3). In contrast, dye was not taken up by untreated roots (Fig. 2IV C) and hence, the root margin remained smooth signifying its functional integrity.

### Biochemical and molecular identification of *Mesorhizobium* and fungicide tolerance

Strain *M. ciceri* was characterized morphologically and biochemically (Table S2). Based on biochemical and cultural characteristics, the genus of the symbiotic bacterium was confirmed and strain was presumed as *Mesorhizobium*. Isolate BRM5 showed the maximum base sequence similarity to strain *Mesorhizobium ciceri*. Based on this relatedness, isolate BRM5 was identified as *Mesorhizobium ciceri*^31^*.*

The tolerance of *M. ciceri* to KITZ was assessed while grown in minimal salt (MS) broth added with variable concentrations of fungicide; strain BRM5 survived up to 2400 µg mL^−1^ of KITZ (Table S2). *Achromobacter spanium* and *Serratia plymuthica* tolerated exceptionally high concentrations of different group of pesticides recovered from pesticide-polluted rhizospheres^59^. Various workers have isolated the pesticide-tolerant bacterium recovered from nodules of different legumes raised in pesticide-polluted soil^60–64^.

### PGP active biomolecules produced by *M. ciceri* under fungicide stress

#### IAA, siderophores and ACC deaminase

Pesticide-tolerant *M. ciceri* revealed inconsistent secretion/production of active biomolecules when cultured in both stressed and controlled (fungicide-free) environments. Under controlled conditions *M. ciceri* synthesized 43.3 ± 3.2 µg IAA mL^−1^ which decreased with increasing doses of KITZ. This strain synthesized indole-3-acetic acid even when cultured in LB broth supplemented with higher concentrations of fungicide. For example, secretion of IAA was reduced from.

**Figure 3:**
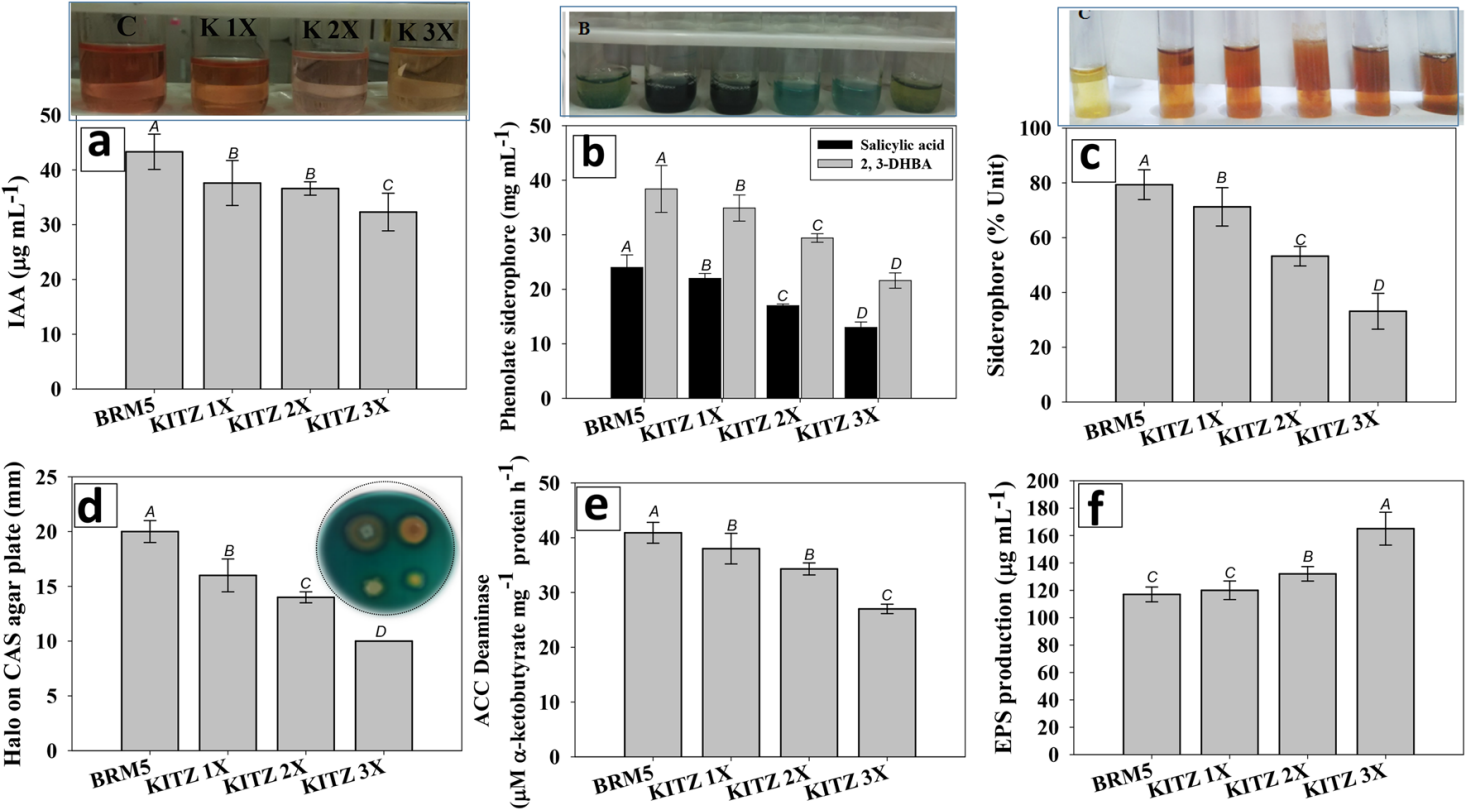
Plant growth regulating bioactive molecules; indole-3-acetic acid (**a**), phenolate type siderophore [salicylic acid and 2.3-DHBA) (**b**), siderophore % unit (**c**), chrome azurol-S agar (**d**), ACC deaminase (**e**) and exopolysaccharide (**f**) produced by *M. ciceri* BRM5 in the absence and presence of different doses of KITZ. In this figure, bar diagrams represents the mean values (mean ± S.D) of three independent replicate whereas, error bars depicts the standard deviation (S.D). Different letters on bars denotes that mean values are significantly different (at *p* ≤ 0.05) according to DMRT.

43.3 ± 3.2 to 32.2 ± 3.1 µgIAAmL^-1^ (25.6% decrease) at 1800 µg KITZ mL^−1^ (Fig. 3a). The impact of different groups of pesticides on IAA and other plant growth regulating active biomolecules of nodule bacterium *Bradyrhizobium* sp.^16^, *B*. *japonicum*^65^ and *R*. *Leguminosarum*^52^ have also been reported. Secretion of IAA by the fungicide-tolerant BRM5 strain even at higher levels of pesticide is a promising feature of soil microbes, because such pesticide-tolerant PGPR strains, when used in harsh environments are likely to endure producing/releasing phytohormones such as IAA. This crucial growth-augmenting plant hormone will thus be accessible to plants even at high levels of pesticides. A trend similar to IAA was observed for siderophore production. *M. ciceri* synthesized siderophores even under stressed conditions (Fig. 3b–d). Similar production of siderophores by *Rhizobium* sp., *Mesorhizobium* sp.^64^, *Pseudomonas* sp.^66^ and *A*. *vinelandi*^62^ under stressed conditions are reported.

Bacterial ACC deaminase is an outstanding biological attribute which can substantially decrease levels of ethylene (C_2_H_2_) in plants and thus, accelerates the functioning of developing plants in harsh environments^67^. *M. ciceri* exhibited a positive response to ACC deaminase even when cultured in fungicide-supplemented medium. Maximum (40.9 ± 1.8 µM α-Ketobutyrate mg^−1^ protein h^−1^) and minimum (27.0 ± 0.87 µM α-Ketobutyrate mg^−1^ protein h^−1^) amounts of ACC were synthesized at 0 and 1800 µg KITZ mL^−1^, respectively (Fig. 3e). Secretion of ACC deaminase by the tolerant strain, however, even in stressed environments is agronomically a beneficial feature and could serve as a promising choice for increasing productivity of crops under pesticide stress.

#### EPS and NH_3_ production

**Figure 4:**
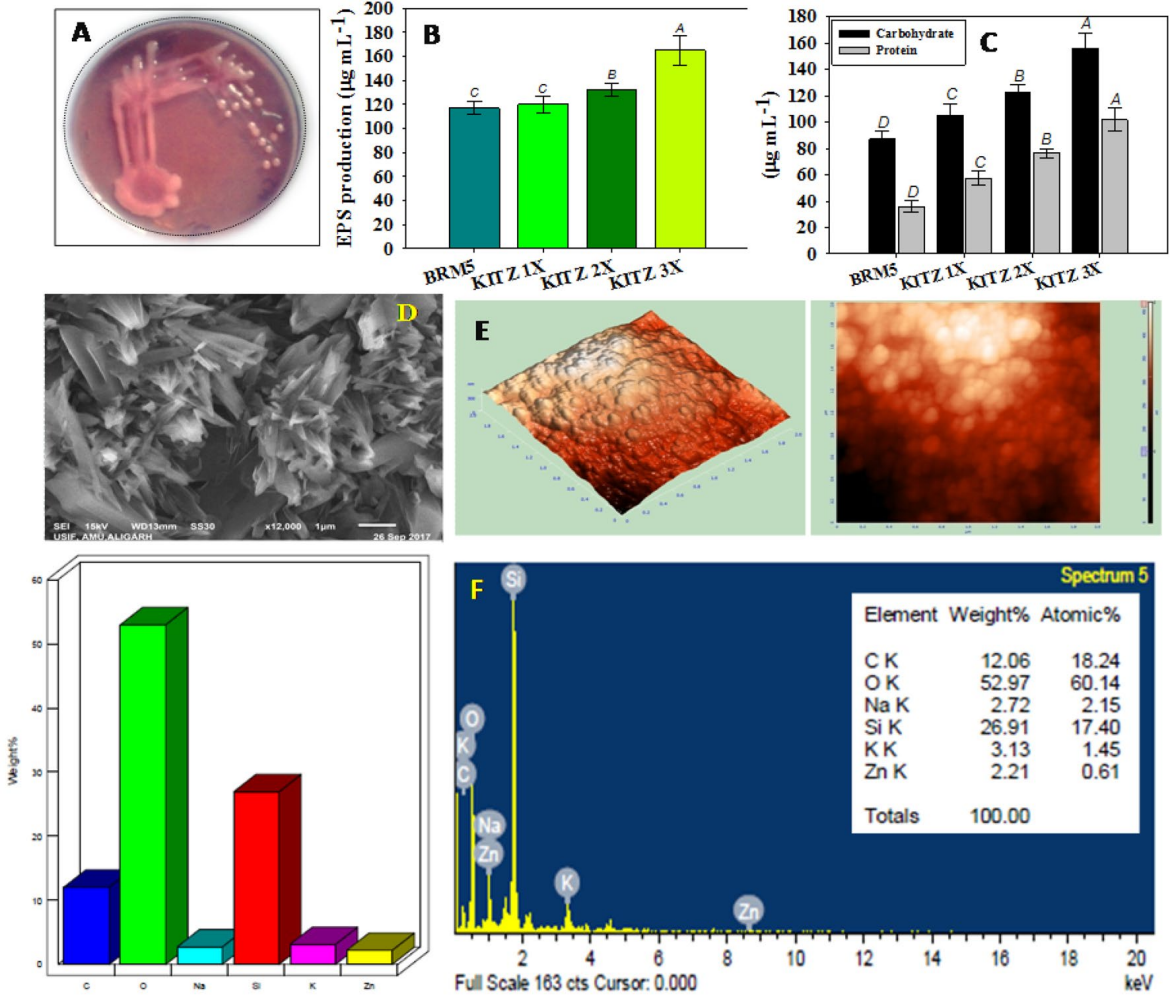
Exopolysaccharide (EPS) producing cultures of *M. ciceri* on YEMA plate (**A**), Bar diagrams represents the EPS synthesized by strain BRM5 in the presence of varying concentrations of KITZ (**B**), quantification of EPS; carbohydrate and protein content (**C**), morphological analysis of dried powder of EPS under SEM (**D**), topographical analysis of dried powder of EPS under Atomic force microscope (AFM) (**E**), EDX analysis of dried powder of EPS showing the presence of various elements (**F**).

EPS synthesized by *M. ciceri* increased with increasing concentrations of fungicide. For example, at 1800 µg KITZ mL^−1^ (3 × dose), *M. ciceri* secreted the maximum EPS which was 29% (165 ± 12 µg mL^−1^) greater than that released under fungicide-free conditions (117 ± 5.4 µg mL^−1^) (Fig. 3f). Furthermore, the EPS secreted by *M. ciceri* BRM5 (Fig. 4A) showed an enhancement with increasing concentrations of FIP (Fig. 4B). Additionally, the EPS produced by strain was quantified (Fig. 4C) using standard protocols. The structural morphology and topography of dried powder of EPS was done using SEM (Fig. 4D) and AFM (Fig. 4E) techniques. Also, the elemental analysis indicated the presence of some major and trace elements (Fig. 4F). Release of EPS by nodule bacterium both in the absence or presence of stressor molecules (pesticides) could be advantageous for producing bacteria and for growing crops. When EPS are liberated from bacterial cells into their surroundings, EPS may influence the growth of plants even under stressed conditions. Exopolysaccharides protects bacteria from desiccation, phagocytosis and phage attack^68^ by forming a polymeric network around growing cells, while it protects plants from pathogen attack^69^. In addition, the rhizobia invasion process, formation of the infection thread, bacteroid, and nodules during *Rhizobium*–legume interactions is greatly influenced by EPS^70^. By synthesizing substantial quantities of EPS, bacterial strains can be safeguarded from the noxious effects of contaminants by masking their effects^71^. Due to these benefits, interest in identifying EPS-producing nodule bacteria has increased in recent years^72^. Similarly, EPS production of PGPR strains under pesticide stress was reported by other workers^73,74^. Bacterial strain showed the activity of ammonia production at all the concentrations of KITZ (Table 1).

**Table 1:** Ammonia, HCN and siderophore production by *M. ciceri* BRM5 under kitazin stressed condition.

Treatment	Dose rate (µg mL^−1^)	NH_3_ production^a^	HCN production^b^	Siderophore production (FeCl_3_ test)
Control	0	++	ND	++
Kitazin	600*	+	ND	+
	1200**	+	ND	+
	1800***	+	ND	+

In this table, *, ** and *** represents the 1 ×, 2 × and 3 × concentrations of kitazin, respectively.

^a^Ammonia.

^b^HCN production.

++ and ND indicate ‘positive reaction’ and ‘not detected’, respectively.

### *C. arietinum*-fungicide-*Mesorhizobium *interactions: comprehensive toxicity and bioremediation studies Bioinoculation impact of *M. ciceri* on Biochemical Characteristics of *C. arietinum*

#### Seed germination

**Figure 5:**
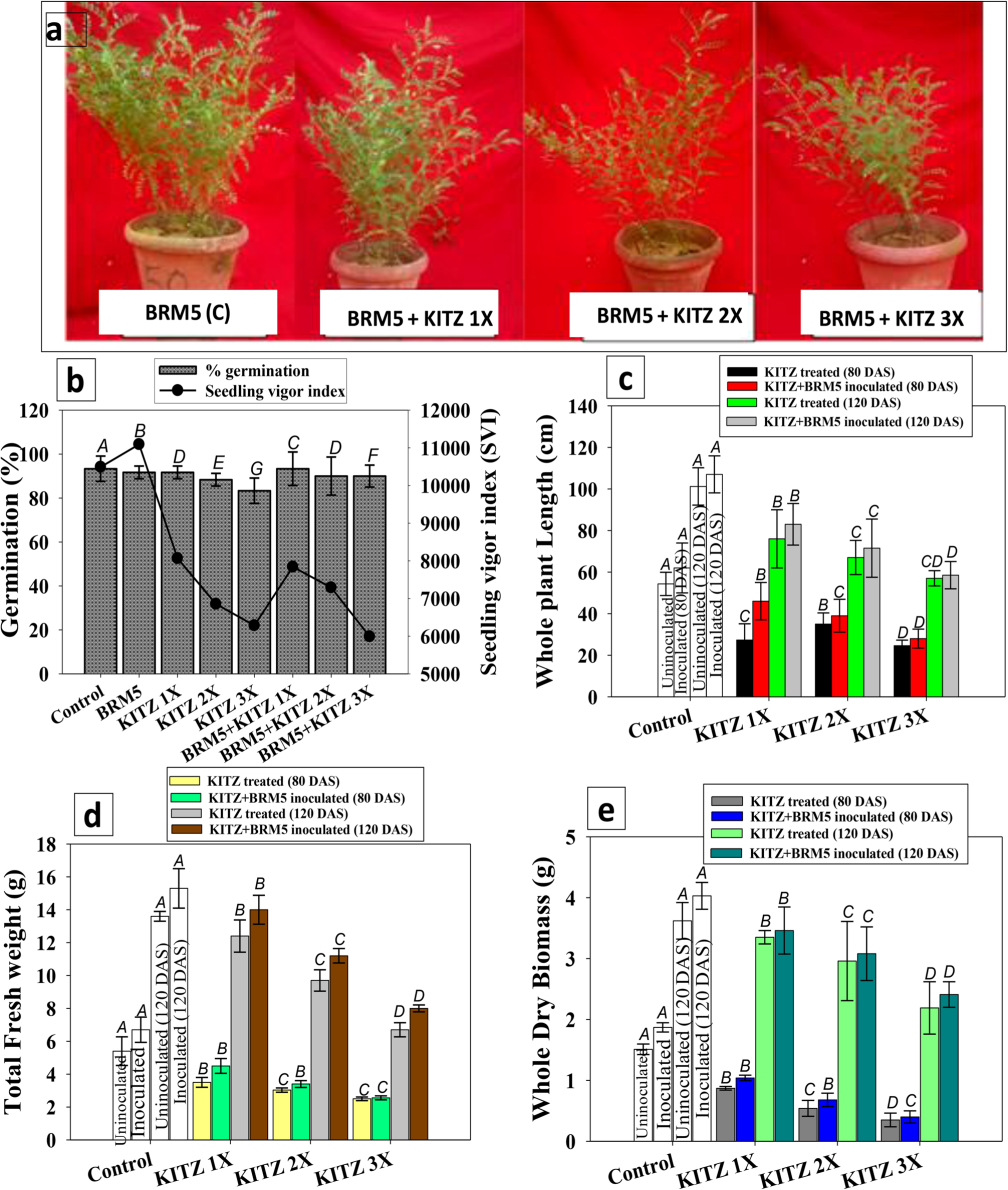
Inoculation impact of kitazin tolerant *M. ciceri* BRM5 on *C. arietinum* plants grown in sandy clay loam soil treated with 96 (1 ×), 192 (2 ×) and 288 (3 ×) µg KITZ kg^−1^ soil developed in greenhouse conditions (**a**), germination efficiency and vigor index (**b**), total plant length (**c**) total fresh weight (**d**) and total dry biomass (**e**). The bar and line diagrams represent mean ± standard deviation (S.D) (*n* = 3) of three replicates where each replicate constituted three plants/pot. Mean values followed by different letters are significantly different at *p* ≤ 0.05 according to DMRT test.

The kitazin tolerant *M. ciceri* improved the growth of plants when applied to *C. arietinum* plants in soil system treated with variable level of fungicide (Fig. 5a). The impact of *M. ciceri* BRM5 on germination efficiency of *C. arietinum* seedlings grown in earthen pots supplemented separately with varying doses of KITZ was variable (Fig. 5b). Generally, strain BRM5 had a positive impact on germination and vigor index relative to un-inoculated seeds. *M. ciceri* BRM5 exhibited a maximum increase of 5% and 6% in germination and SVI at 96 µg KITZ kg^−1^ (Fig. 5b) compared to the un-inoculated but similar dose of fungicide. *Microbacterium hydrocarbonoxydans* BHUJP-P1, *Stenotrophomonas rhizophila* BHUJP-P2, *B. licheniformis* BHUJP-P3 and *B. cereus* BHUJP-P4 increased germination efficiency and growth of crops even in the presence of varying concentrations of monocrotophos and chlorpyrifos^27^.

#### Length and weight of plant organs

A gradual increase in length of roots and shoots of *M. ciceri* BRM5-bacterized plants was observed (Fig. 5c) both at 80 and 120 DAS in the presence of variable doses of KITZ. *M. ciceri* BRM5, when grown with KITZ, imparted maximum benefits on plant organs which decreased considerably at 3 × concentration. *M. ciceri* BRM5 at 96 µgKTZkg^-1^ increased the lengths of roots, shoots and whole plants maximally by 11, 7 and 41% (at 80 DAS) respectively, over sole application of 1 × of KITZ (Table S3, Fig. 5c). Similarly, *M. ciceri* BRM5 increased the fresh weight of roots, shoots and whole plant by 29, 18 and 22%, respectively (120 DAS) when used with 96 µg KITZ kg^−1^ compared to un-inoculated plants at the identical dose of KITZ (Table S3, Fig. 5d). Enhancement in plant growth may be due to increased availability of IAA, soluble P, siderophores, and ACC deaminase secreted by the microbial symbiont. Of these, growth regulators like IAA promote root growth directly by stimulating cell elongation or cell division^75^. The well-developed and expanded roots absorb more water and minerals from soil^76^ which in turn enhance the growth of the plant. Kumar et al*.*^77^ reported that pesticide-tolerant PGPR strains *P. putida* and *B. amyloliquefaciens* alleviated the adverse effect of pesticides and increased soil enzyme activities and seed germination efficiency, elongated plant organs and enhanced other parameters of *C. arietinum*. These findings corroborate our facts that inoculated PGPR degrade/detoxify pesticides and thus, improve various parameters of legumes grown in the affected soil.

#### Dry biomass accumulation

The bacterized and un-inoculated *C. arietinum* plants cultivated in soil treated with varying levels of fungicide had variable dry biomass of *C. arietinum*. A gradual increase in root and shoot biomass of *M. ciceri* BRM5-inoculated plants treated with different doses of KITZ was observed both at 80 and 120 DAS. *M. ciceri* BRM5 maximally increased root, shoot and total dry biomass by 12, 17 and 18%, (at 80 DAS) when applied in soil treated with 96 µg KITZ kg^−1^ compared to dry biomass of un-inoculated but treated with the same dose of KITZ (Table S3, Fig. 5 e). There was a significant (*p* ≤ 0.05) interaction between applications of symbiotic bacterium and fungicide. The effect of bio-priming and fungicide on biological and chemical characteristics of test plants was significantly correlated both at 80 DAS and 120 DAS as revealed by regression analysis and principal component analysis (PCA) (Figs. S1 and S2).

#### Bio-inoculation impact of *M. ciceri* on photosynthetic molecules

The effect of varying concentrations of KITZ on photosynthetic pigments of *C. arietinum* in the presence of *M. ciceri* BRM5 at 80 DAS was variable. On comparing the effect of the 1 × concentration of KITZ on inoculated and non-inoculated plants, a maximal increases of 14% (0.28 mg g^−1^), 11% (0.28 mg g^−1^), 5% (0.40 mg g^−1^) and 9% (1.21 mg g^−1^) in Chl a, Chl b, total chlorophyll and carotenoids content, respectively, was noted in *M. ciceri* BRM5-inoculated *C. arietinum* plants over untreated and non-inoculated control plants (Table S3). Likewise, photosynthetic pigments of *C. arietinum* plants improved following the inoculation of pesticide-tolerant PGPR strains. In this context, enhanced symbiotic performance and productivity of *Phaseolus vulgaris* in harsh environmental conditions was observed following the inoculation of tolerant native PGPR^78^. Increased nodulation and seed yield in *V. faba* due to inoculation of *Rhizobium* has been reported^79^.

#### Effect of *M. ciceri* on symbiotic features of *C. arietinum*

##### Nodulation: nodule numbers and nodule dry biomass

**Figure 6:**
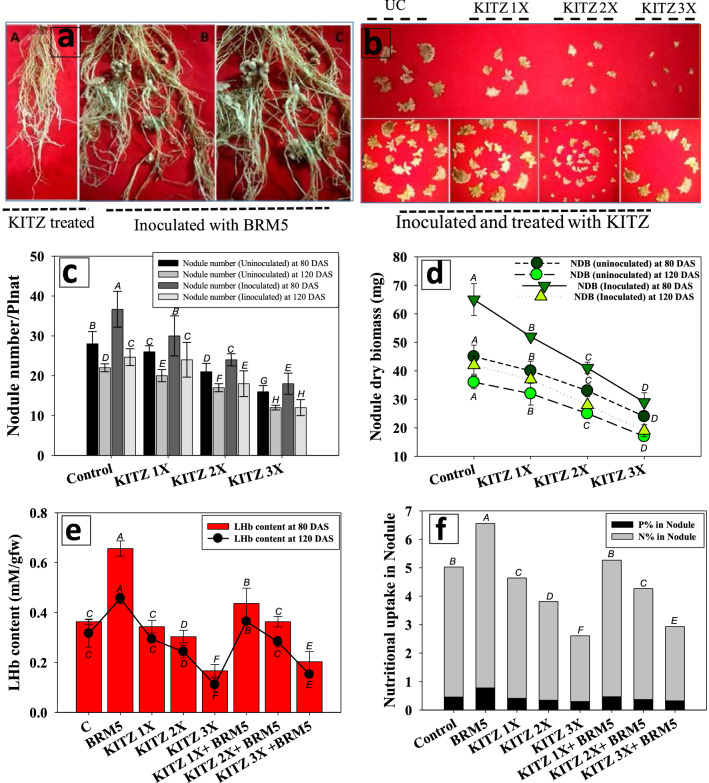
Bio-inoculation impact of *M. ciceri* BRM5 on symbiotic features of *C. arietinum* plants; attachment of nodules with inoculated and treated roots (**a**), morphology of nodule (**b**) nodule number (**c**), nodule dry biomass (**d**) and LHb content (**e**) and nutrient uptake in nodules (**f**) grown in sandy clay loam soil treated with 96 (1 ×), 192 (2 ×) and 288 (3 ×) µg KTZ kg^−1^ soil and harvested at different intervals. The bar and line diagrams represent the mean ± S.D (*n* = 3) of three replicates where each replicate constituted three plants/pot. Mean values followed by different letters are significantly different at *p* ≤ 0.05 according to DMRT test.

The roots detached from un-inoculated and KITZ treated *C. arietinum* showed the poorly developed root system and weak/unhealthy nodular systems. In contrast, a better root system having healthy and more pink-colored showing wavy margin was recorded in bio-inoculated *C. arietinum* plant (Fig. 6a,b). Generally, the symbiotic attributes [nodule number (NN) and nodule dry biomass (NDB)] of *M. ciceri* BRM5 bacterized *C. arietinum* plants grown in the presence of KITZ was greater compared to those recorded for un-inoculated plants supplemented with the identical dose of fungicide. *M. ciceri* BRM5 maximally increased NN and NDB by 23% and 22% at 80 DAS and 16% and 14%, respectively at 120 DAS when used with 96 µg KITZ kg^−1^ compared to un-inoculated but KITZ-treated plants (Fig. 6c,d). Ullah et al*.*^80^ also reported that PGPR strains in combination with *M. ciceri* increased the growth and nodulation of *C. arietinum* even in pesticide-stressed conditions.

##### Leghaemoglobin (LHb) content and nutrient uptake in nodule

The LHb content in fresh nodules of *C. arietinum* declined in the presence of symbiotic bacteria similar to that in plants grown in fungicide only-supplemented soils. A considerable improvement in LHb content of bacterized plants was, however, observed at 80 DAS relative to plants grown in soil treated only with pesticide. *M. ciceri* BRM5 significantly (*p* ≤ 0.05) and maximally enhanced LHb content by 21% [(0.34 to 0.43 mM (gfw)^−1^] at 96 µg KITZ kg^−1^ over LHb content of un-inoculated but KITZ-treated plants (Fig. 6e). Furthermore, the nutritional uptake in nodule was assessed was and it was recorded that N and P was maximally increased by *M. ciceri* when root systems were detached from plants treated with 1 × dose of KITZ (Fig. 6f).

#### Impact of *M. ciceri* on grain attributes and nutrient uptake of *C. arietinum*

##### Seed yield (SY) and grain protein

**Figure 7:**
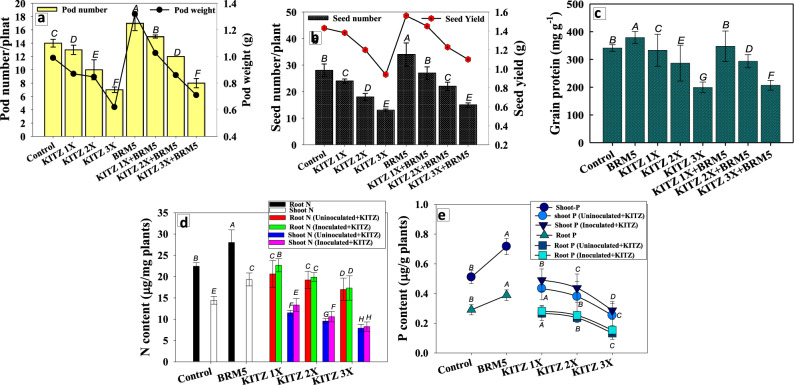
Bio-inoculation impact of *M. ciceri* BRM5 on *C. arietinum* plants on: pod number and yield (**a**), seed number and seed yield (**b**), grain protein (**c**), N content (**d**) and P content (**e**) grown in sandy clay loam soil treated with 96, 192 and 288 µg KTZ kg^−1^ soil. The bar and line diagrams represent the mean ± S.D (*n* = 3) of three replicates where each replicate constituted three plants/pot. Mean values followed by different letters are significantly different at *p* ≤ 0.05 according to DMRT test.

Number of seeds, yield and grain protein of *C. arietinum* declined significantly in the presence of varying doses of KITZ. In contrast, seed yield (SY) of *M. ciceri* BRM5-inoculated *C. arietinum* plants improved significantly (*p* ≤ 0.05) in the presence of different doses of KITZ. A maximum increase of 13%, 15%, 11% and 6% in pod number, pod weight, seed number and seed yield was recorded when *M. ciceri* BRM5 was used with 96 μg KITZ kg^-−1^ soil over un-inoculated but pesticide-treated control (Fig. 7a,b). Likewise, BRM5 increased the grain protein by 7% when used with the 1 × dose of KITZ compared to un-inoculated plants treated with the identical dose of fungicide (Fig. 7c). The decrease in protein content of grain is likely be due to the binding of pesticides to R-SH groups of proteins which, in turn, alters protein structure. However, seed features of *C. arietinum* were generally improved following inoculation with *M. ciceri* BRM5 even in the presence of pesticide. *R. leguminosarum* strain PS1, when used as bio-inoculant in pesticide-treated pea plants, increased SY by 43% compared with fungicide-treated but un-inoculated plants^81^. Enhancement in growth attributes and yield of atrazine-treated *Phaseolus vulgaris* when grown in the presence of a consortium containing *Rhizobium* sp*.* and *Trichoderma* have been reported^82^. Similar improvements in growth and nutrient levels were observed when stress-resistant PGPR strains of *P. aeruginosa and Burkholderia gladioli* were applied to plants grown under stressed conditions^83^.

##### Nutrient uptake

Bio-inoculation impact of *M. ciceri* BRM5 on N and P content of *C. arietinum* plant organs at 80 DAS differed in a concentration-dependent manner. The N content in *C. arietinum* roots increased from 20.6 to 22.6 µg g^−1^ whereas in shoot tissue it increased from 11.5 to 13.3 µg g^−1^ when *M. ciceri* BRM5 was used in the presence of 96 μg KITZ kg^−1^ (Fig. 7d). Similarly, *M. ciceri* BRM5 at 96 μg KTZ kg^−1^ soil improved root and shoot P by 7 and 11%, respectively, compared to non-inoculated plants treated with similar concentrations of KTZ (Fig. 7e). Uptake of nutrients (N and P) by *C. arietinum* raised in pesticide-enriched soils was also enhanced following inoculation with fungicide-tolerant symbiotic bacteria. A similar pattern in growth and uptake of major and trace nutrients viz., N, P, K, Ca, S and Fe in different varieties of PGPR inoculated legumes raised in stressed soils was reported^84^. In another study, *Phaseolus vulgaris* plants inoculated with stress-tolerant PGPR belonging to a group of phosphate-solubilizing bacteria significantly lowered electrolyte leakage, LPO level, SOD, hydrogen peroxide and proline phosphatase activities and improved physio-biochemical attributes, nutrient uptake, and protein and carbohydrate content by relieving the stress^85^.

#### Proline, MDA and antioxidant enzymes

##### Proline and MDA content

**Figure 8:**
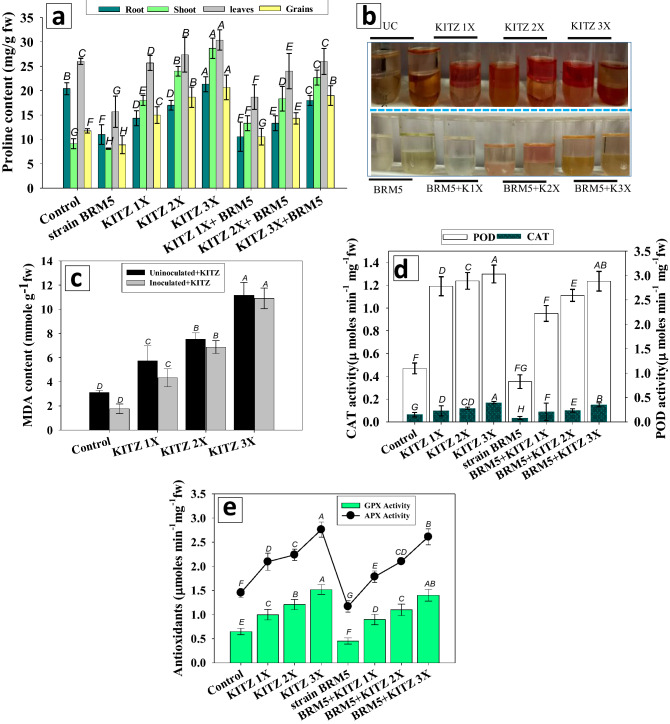
Bio-inoculation impact of *M. ciceri* BRM5 on proline content (**a**) antioxidant enzymes: GPX (**b**), APX (**c**) CAT (**d**) and MDA content (**e**) of *C. arietinum* plants grown in sandy clay loam soil treated with 96 (1 ×), 192 (2 ×) and 288 (3 ×) µg KTZ kg^−1^ soil. The bar and line diagrams represent the mean ± S.D (*n* = 3) of three replicates where each replicate constituted three plants/pot. Mean values followed by different letters are significantly different at *p* ≤ 0.05 according to DMRT test.

The BRM5-inoculated *C. arietinum* plants had low levels of proline, MDA and antioxidant enzyme activity in organs even in the presence of different concentrations of KITZ. In general, strain BRM5 minimized the proline level even in the presence of fungicide. *M. ciceri* BRM5 significantly (*p* ≤ 0.05) and maximally reduced the proline content in roots, shoots and seeds by 27%, 26% and 33% at the 1 × dose of KITZ (Fig. 8a,b) compared to un-inoculated plants treated with the identical dose of fungicide. Similarly, it was observed that *M. ciceri* BRM5 reduced the MDA content from 5.4 to 3.07 µ moles g^−1^ fw compared to un-inoculated plants treated with 96 μg KITZ kg^−1^ (Fig. 8c). Information is lacking as to how and why proline levels decline in legumes bio-inoculated with pesticide-tolerant PGPR and raised in pesticide-contaminated soil. The accumulation of proline declined significantly (*p* ≤ 0.05) in bio-primed *C. arietinum* grown in soil supplemented with high doses of pesticide. A similar reduction in proline content in foliage was recorded following inoculation of three phosphate-solubilizing strains of *Pseudomonas* (*Pseudomonas* sp. *P. putida* and *P. fulva*) under chlorpyrifos and pyriproxyfen stress^86^. The bio-inoculation impact on MDA level in the presence of higher doses of KITZ varied considerably among the bacterial species. In another study, inoculation of plants with *P. putida* caused substantial reduction in lipid peroxidation biomarkers, MDA content and electrolyte leakage and gradually improved chlorophyll, carotenoid and carbohydrate contents and growth of plants under norflurazon (herbicide)-stressed conditions^87^. Fungicide-tolerant *M. ciceri* BRM5 used in this study resulted in a significant decrease in MDA concentrations in pesticide-stressed *C. arietinum* plants. The inoculant ameliorated pesticide pressure, relieved oxidative damage in plants and allowed them to grow pesticide-polluted soil. These results indicate that the bacterial strains could serve in bioremediation strategies which lead to overall improvement in performance of leguminous crops while being cultivated under pesticide stress.

##### Antioxidant enzymes

Antioxidant enzymes of *C. arietinum* foliage treated with different doses of KITZ decreased substantially in the presence of bacterial inoculants. CAT activity of *C. arietinum* foliage was reduced maximally and significantly (*p* ≤ 0.05) by (8.3%) by *M. ciceri* BRM5 in the presence of 96 KTZ μg kg^−1^ soil (Fig. 8d). Similarly, bacterial strains maximally lowered the POD activity by 21% (from 2.78 to 2.2 µ mol min^−1^ mg^−1^ fw) in the foliage system when detached from 1 × concentration of KITZ (Fig. 8d). Likewise, the APX and GPX activities of *C. arietinum* were increased by 19.6% (from 2.09 to 1.68 µ mol min^−1^ mg^−1^ fw and 9% (from 0.99 to 0.90 µ mol min^−1^ mg^−1^ fw respectively at the 3 × dose of KITZ following application of symbiotic bacterium (Fig. 8e). In a similar study, strain SRB02 of *B. aryabhattai* considerably reduced levels of oxidative stress and antioxidant enzymes CAT, POD and SOD in soybean plants grown in stressed soil, and promoted overall growth of plants^88^. The declines in antioxidants due to inoculation with pesticide-tolerant bacterial strains consequently resulted in a substantial upsurge in overall growth of *C. arietinum* even under pesticide stress.

#### Rhizosphere and rhizoplane colonization by *M.ciceri* under fungicide

**Figure 9:**
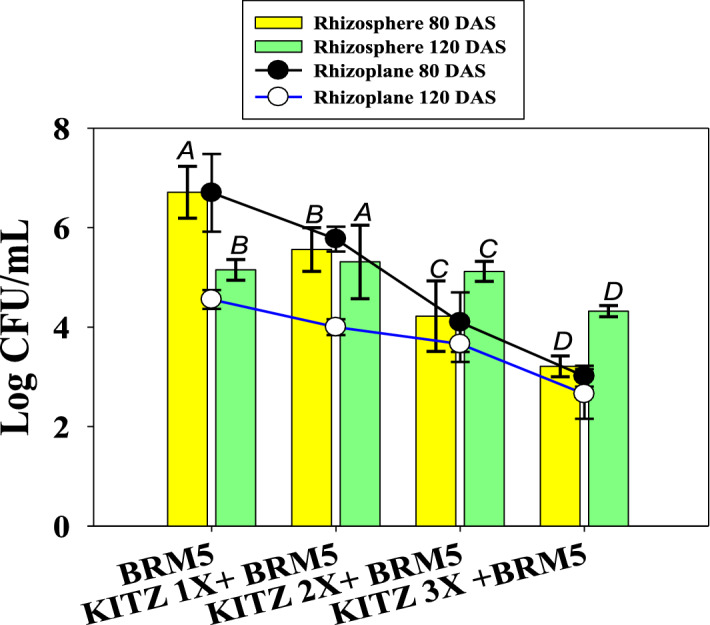
Rhizoplane and rhizosphere colonization in the presence of different concentrations of kitazin at two different seeding stages (80 and 120 DAS).

Root colonization is an important factor in plant–microbe interactions. The resulting mutualistic interaction is considered helpful in growth and development of plants as well as in protecting the crops from various biotic and abiotic stresses^89^. Considering this, halotolerant PGPR strain *M. ciceri* was checked for its root colonizing ability using SEM in the absence (Fig. S3A) and presence of KITZ (Fig. S3B). SEM images revealed that fungicide-untreated roots resulted in dense/compact colonization whereas treated roots showed lesser bacterial populations. Similar colonization of bacteria on *C. arietinum* root surfaces and consequent increase in plant growth was reported by other workers^90–92^. Bacterial components such as EPS, cell wall polysaccharides and extracellular bacterial proteins help bacteria to attach onto the root surface. Bacterial counts from the rhizosphere and rhizoplane at varying intervals of seeding, i.e., 80 and 120 DAS with different doses of KITZ, were determined. The CFU counts were significantly reduced over untreated control with increasing level of fungicide; however, strain BRM5 survived and colonized even at the higher dose of fungicide. The lower doses imparted lesser impacts on viable counts of bacteria. At 3 × KITZ, the rhizospheric CFU count of *M. ciceri* was 3.2 and 4.3 log CFU/mL as compared to 6.7 and 5.1 log CFU/mL (untreated control) at 80 DAS and 120 DAS, respectively (Fig. 9). Similarly, viable populations of rhizoplane bacteria had declined over the untreated control. As PGPR colonize root surfaces, they multiply and reproduce by receiving key signaling compounds and nutrients from root exudates which subsequently leads to biofilm formation on the root system, a prominent indicator of successful plant–microbe interaction.

## Conclusion

Fungicide applied in the study detrimentally affected the germinative ability of *C. arietinum* leading to the decrease in plant growth parameters as recorded both under in vitro and soil system. The *M. ciceri* applied in this study could tolerate higher level of kitazin and synthesized many growth regulating bioactive molecules even in fungicide supplemented media. Following application to soil, *M. ciceri* improved the performance of *C. arietinum* and enhanced dry biomass production, yield, symbiosis and leaf pigments even in a fungicide-polluted environment. Additionally, pesticide-tolerant nodule bacterium *M. ciceri* declined the stressor metabolites and antioxidant status of plants. The present study creates a new perspective for understanding the mechanistic basis of declines in stressor molecules and antioxidant defense enzymes in symbiotic bacterium-inoculated *C. arietinum* grown in fungicide-contaminated soil. Furthermore, symbiotic strain effectively colonized the plant rhizosphere/rhizoplane. Conclusively, under pesticide- contaminated soils, inoculation of *M. ciceri* may serve as an excellent microbial strategy for augmenting *C. arietinum* productivity.

## Supplementary Information

Supplementary Information.

## Data Availability

The datasets generated during and/or analyzed during the current study are available from the corresponding author on reasonable request.
