# Supplementary material for: Mesorhizobium ciceri as biological tool for improving physiological, biochemical and antioxidant state of Cicer aritienum (L.) under fungicide stress
Source: Sci Rep. 2021 May 6;11:9655. doi: 10.1038/s41598-021-89103-9 (PMC8102606; doi:10.1038/s41598-021-89103-9)
Supplement: Supplementary file 1 — Supplementary Information. [file 41598_2021_89103_MOESM1_ESM.docx]

**Electronic Supporting Information**

***Mesorhizobium ciceri* as Biological Tool for improving Physiological, Biochemical and Antioxidant state of *Cicer aritienum* (L.) under fungicide stress**

***Mohammad Shahid*^a^*, Mohammad Saghir Khan*^a^*, Asad Syed*^b^*, Najat Marraiki*^b^*, Abdallah M. Elgorban*^b^***

*^a^*Department of Agricultural Microbiology, Faculty of Agricultural Sciences, Aligarh Muslim University, Aligarh-202002; Uttar Pradesh, India

***^b^***Department of Botany and Microbiology, College of Science, King Saud University, P.O. Box 2455, Riyadh 11451, Saudi Arabia

*Correspondence to:

**Mohammad Shahid**

**E-mail:** [shahidfaiz5@gmail.com](mailto:shahidfaiz5@gmail.com)

Department of Agricultural Microbiology,

Faculty of Agricultural Sciences,

Aligarh Muslim University, Aligarh, India

+91-08090939511

**Supplementary Methods**

***2.1 Toxicity Assessment of Fungicide under In-Vitro***

***2.1.1 Germination percentage and Seedling vigor index (SVI)***

Healthy chickpea seeds were surface sterilized using 3% (w/v) sodium hypochlorite (NaOCl) for three min. followed by three successive washing with distilled water (DW). Five days after sowing (DAS), radicle emergence of one millimetre was considered as germinated seeds and germination percentage was calculated as:

Germination % = Number of seeds germinated ×100

Total number of seeds

The percentage of germination was used further to calculate the seedling vigor index

Seedling vigor Index (SVI) = [RL+ SL] × % Seed Germination

Where, RL= Root length, SL= Shoot length

***2.1.2 Percent phytotoxicity, Tolerance Index (TI) and Root shoot length Ratio***

The percentage of phytotoxicity was calculated according to Chou and Lin (1976) as:

% Phytotoxicity = Shoot or root length of control − Shoot or root length of treatment ×100

Shoot or root length of control

Where, RL and SL are root and shoot length, respectively.

Tolerance index (TI) was determined by the formula used by Iqbal and Rahmati (1992).

Tolerance indices (TI) = RL of fungicide treatment ×100

RL of control treatment

***2.4 Identification of BRM5 using 16SrRNA gene sequencing***

The total genomic DNA was extracted by the method as previously described by Yadav et al. (2011). The forward primer pA (5’AGA GTT TGA TCC TGG CTC AG3’) and reverse primer pH (5’AAG GAG GTG ATC CAG CCG CA3’) (Solanki et al. 2012) were used to amplify the 16S rRNA gene from genomic DNA. The total volume of reaction mixture was 100 µl containing 50–80 ng of template DNA, 10X reaction buffer, 2.5mM dNTPs, 20 pM of each primer and one unit Taq DNA polymerase (Bangalore Genei, India), and reactions were performed on G-storm thermocycler (G-STORM, UK). The amplification conditions were as follows: initial denaturation at 94°C for 5 min, followed by 35 cycles of denaturation at 94°C for 40 s, annealing at 52 °C for 45 s, and elongation at 72 °C for 1min 30s. At the end of 35 cycles, the final extension step was at 72 °C for 8 min. The amplified product was resolved by electrophoresis in 1.2% agarose gel in 1X TAE buffer. Gels were stained with ethidium bromide (10 mg ml^-1^) and visualized on gel documentation system (BIO-RAD, USA). Strong and clear bands were scored for similarity and clustering analysis using the software, NTSYS-2.02e package (Numerical taxonomy analysis program package, Exeter software, USA).The purified16S rRNA PCR products were send to Macrogen, Seol, south Korea for 16S rRNA sequencing. The 16S rRNA amplicons were sequenced from both ends and consensus sequence was generated. The partial 16S rRNA gene sequences were compared with those available in the databases (http://www.ncbi.nlm.nih.gov/BLAST/) and identification to the species level was determined on the basis of sequence similarity of >97% with the closest relative in the GenBank. The phylogenetic tree was constructed on the aligned datasets using the neighbour-joining method implemented in the program MEGA 4.0.2 (Tamura et al. 2007). Bootstrap analysis was performed on 1,000 random samples taken from the multiple alignments.

***2.5.2 HCN and NH_3_ production***

HCN production by strain BRM5 was evaluated by the method of Bakker and Schippers (1987). For this, strains were inoculated on HCN induction (King’s B agar plates amended with 4.4 g L^-1^ glycine) medium supplemented with three concentrations of KITZ and incubated at 28±2 ^0^C for 4 days. A disk of Whatman filter paper No. 1 soaked in 0.5% picric acid and 2% Na_2_CO_3_ was placed under the lid of the petri plates and sealed with parafilm. After 4 days of incubation at 28±2 ^0^C, an orange-brown colour of the paper confirmed the production of cyanogenic compound (HCN). The NH_3_ produced by strain was detected by growing the bacterial cells (100 µL) in 10 mL of peptone water (g L^-1^: peptone 10 g, NaCl 5 g, pH=7) amended with control (0), normal (1X) double (2X) and three times more (3X) rates of each pesticide and incubated at 28±2°C for 4 days. After incubation, Nessler reagent (1 mL) was added to each tube and the development of yellow colour showed ammonia production (Dye, 1962).

***2.6.2 Bio-inoculation Impact of Mesorhizobium on photosynthetic pigments of C. aritienum foliage***

Photosynthetic pigments (Chl a, Chl b, total chlorophyll and carotenoid content) in fresh foliage of bio inoculated chickpea grown in soil treated with varying doses of KITZ was measured according to the method of Arnon (1949). The pigments were extracted from fresh leaves by macerating in 80% acetone. Absorption of chlorophyll and carotenoid content in the extract was determined using UV visible spectrophotometer (UV-2450, Shimadzu, Tokyo, Japan). The total photosynthetic pigments (Chl a, Chl b and total chlorophyll) was calculated as:

mg chl. a/g tissue = 12.7 (A_663_) −2.69 (A_645_) ×V/1000×W

mg chl. b/g tissue = 22.9 (A_645_) − 4.68 (A_663_) ×V/1000×W

mg total chl./g tissue = 20.2 (A_645_) +8.02 (A_663_) ×V/1000×W

Carotenoid content was determined by the formula as suggested by Krik and Allen (1965):

Carotenoids (mg/g tissue) = (A_480_) +0.114 (A_663_) – 0.638 (A_645_)

Where, A_λ_ = absorbance at specific wavelength λ (nm); V= final volume of chlorophyll extracted in 80% acetone, and W= fresh weight of tissue extract.

***2.6.3 LHb content Estimation***

Briefly, fresh nodules were crushed with the help of mortar and pestle in a 5 mL phosphate buffer (pH=7.4) and filtered through two layers of cheese cloth. The turbid reddish-brown filtrate was clarified by centrifugation at 10000 g for 30 min. The supernatant was diluted to 10 mL with phosphate buffer (pH=7.4). The extract was divided equally into two glass tubes (5 mL/tube) and an equal amount of alkaline pyridine reagent was added to each tube. The haemachrome formed was read at 556 and 539 λ after adding a few crystals of potassium hexacyanoferrate (K_3_FeCN_6_) and sodium dithionite (Na_2_S_2_O_4_), respectively. The LHb content was calculated using the formula:

LHb content (mM) = [λ_556_-λ_539_] × 2D/23.4

Where, D= initial dilution

***2.6.3 Nutrient uptake and seed features in M. ciceri inoculated and fungicide treated C. arietinum plants***

For protein estimation, 500 mg of seeds were soaked in phosphate buffer (pH=7.4) and extracted in 3 mL of 50 mM phosphate buffer (pH=7.8) containing 1mM EDTA and 2% w/v polyvinylpyrrolidone (PVP). The extract was spun at 5742 g for 10 min. at 4°C and supernatant was used for protein analysis. A- 0.2 mL aliquot was taken from the sample extract and the volume was made up to 1.0 mL. To it, 4.5 mL of copper solution was added and was allowed to stand for 10 min. Then, 0.5 mL of Folin’s reagent was added to each tube and incubated for 30 min. for colour development. Absorbance of blue colour was read at 660 nm on a UV-Vis spectrophotometer. The protein concentration in the supernatant was determined using a calibration curve of BSA as a standard.

***2.7.1 Estimation of Proline***

Briefly, one-gram of fresh plant organ was homogenized with 5 mL of 3% (w/v) aqueous sulfosalicylic acid (C_7_H_6_O_6_S). The resulting homogenate was filtered through Whatman No.2 filter paper. The resulting cell extract was then centrifuged at 8000 rpm for 20 min, to remove cell debris. The cell filtrate (2 mL) with free proline was treated with 2 mL of acid ninhydrin and glacial acetic acid (2 mL) at 80 °C for one h. The mixture was heated in boiling water bath for one hour. The reaction was terminated by placing the tubes in ice bath. A- 4 mL of toluene was added to the reaction mixture and stirred well for 20 to 30 seconds. Coloured complex was extracted in toluene and the toluene layer was separated. The red colour intensity was measured at 520 nm. A series of standard with pure proline was run in a similar way by dissolving proline in 3% C_7_H_6_O_6_S and a standard curve was prepared. Amount of free proline in the test sample was determined from the standard curve. Proline content on fresh weight basis (μ moles per gram of fresh weight of tissue) was expressed as:

$$Proline content =\frac{\mu gproline/ ml\times mloftoluene}{115.5}\times\frac{5}{gofsample}$$

Where, 115.5 is the molecular weight of proline.

**Supplementary Tables**

**Table S1:** Characteristic features of Kitazin

| **Characteristics** | **Kitazin** |
| --- | --- |
| Common name | Kitazin |
| Chemical name | O, O-Bis(1-methylethyl)S-phenylmethylphosphorothioate |
| Chemical family | Organophosphate |
| Grade | Commercial(48% EC) |
| Trade name | Kitazin |
| Recommended dose | 96 µg/kg |
| Appearance | transparent liquid |
| Molecular weight | 288.344 g/mol |
| Empirical formula | C_13_H_21_O_3_PS |
| Solubility | Water/DMSO |
| Source | P.I. Industries Ltd., Rajasthan, India |

**Table S2:** Microbiological, morphological, biochemical and molecular features of *Mesorhizobium ciceri* strain BRM5

| **Characteristics** | ***Mesorhizobium ciceri* BRM5** |
| --- | --- |
| [A] *Microbiology*  [B] *Morphology* | Gram negative, short rods  White pinkish, mucilaginous, raised, semi translucent colony |
| [C] *Biochemical reactions* |  |
| 1. Citrate utilization | ˗ |
| 1. Indole | ˗ |
| 1. Methyl red | + |
| 1. Nitrate reduction | + |
| 1. Oxidase | + |
| 1. Catalase | + |
| (vii) Voges-Proskauer | ˗ |
| [C.1] *Carbohydrate utilization* |  |
| 1. Glucose | + |
| 1. Lactose | + |
| 1. Fructose | + |
| 1. Sucrose 2. Arabinose 3. Dextrose 4. Maltose | +  +  +  + |
| [C.2] Hydrolysis |  |
| 1. Starch | + |
| 1. Gelatin | + |
| [D] Tolerance to kitazin | Solid (μg mL^-1^) Liquid (μg mL^-1^)  2400 1500 |
| [E] Molecular characteristics  (i) Nucleotide base pair (bp)  (ii) Primer used  (iii) GenBank Accession No.  (iv) Sequences submitted to | 614 bp  785F (5’GGATTAGATACCCTGGTA-3’ and  907R (5’CCGTCAATTCMTTTRAGTTT-3’)  KY013481  NCBI data bank |

**Table S3:** Inoculation impact of *Mesorhizobium ciceri* BRM5 strain on biological attributes and photosynthetic pigments of chickpea grown in sandy clay loam soil treated with/without agrochemical kitazin

| **Treatment** | **Dose rate (μg kg^-1^ soil)** | **Plant length (cm/plant)** | | | | **Fresh weight (g/plant)** | | | | **Dry biomass (g/plant)** | | | | **Photosynthetic pigments**  **(mg/g fw)** | | | |  |
| --- | --- | --- | --- | --- | --- | --- | --- | --- | --- | --- | --- | --- | --- | --- | --- | --- | --- | --- |
|  |  | **Shoot** | | **Root** | | **Shoot** | | **Root** | | **Shoot** | | **Root** | | **Chl a** | **Chl b** | **Total Chl** | **Carotenoid** |  |
|  |  | **80 DAS** | **120 DAS** | **80 DAS** | **120 DAS** | **80 DAS** | **120 DAS** | **80 DAS** | **120 DAS** | **80 DAS** | **120 DAS** | **80 DAS** | **120 DAS** |  |  |  |  |  |
| **Uninoculated** | 0 | 35.3b | 63.6b | 19b | 37.6b | 3.5bc | 9.7d | 1.9c | 3.9b | 1.3d | 3.2a | 0.21b | 0.42b | 0.31b | 0.29c | 0.42b | 1.25b |  |
|  | 96 | 26.3c | 41.0c | 16d | 35.0c | 2.3b | 8.8c | 1.2c | 3.6b | 0.72c | 3.0b | 0.15c | 0.35c | 0.24c | 0.26a | 0.38c | 1.1c |  |
|  | 192 | 20.0d | 36.0e | 15e | 31.0d | 2.03c | 6.9e | 1.0d | 2.8d | 0.45e | 2.7c | 0.09d | 0.26d | 0.21d | 0.16e | 0.36d | 1.0d |  |
|  | 288 | 15.6f | 32.0f | 09f | 25.0e | 1.68e | 4.5g | 0.82f | 2.2f | 0.29f | 2.0d | 0.06f | 0.19e | 0.17e | 0.15e | 0.23f | 0.9e |  |
| **Inoculated** | 0 | 39.0a | 66.0a | 23a | 41.0a | 4.6a | 11.0b | 2.1a | 4.3a | 1.63a | 3.4a | 0.24a | 0.63a | 0.4a | 0.32b | 0.57a | 1.36a |  |
|  | 96 | 28.0c | 45.0c | 18c | 38.0b | 2.8a | 10.1a | 1.7b | 3.9a | 0.87b | 3.1b | 0.17c | 0.36c | 0.28c | 0.28a | 0.40c | 1.21c |  |
|  | 192 | 22.0d | 38.d | 17d | 33.5d | 2.2c | 8.2d | 1.2c | 3.0c | 0.57d | 2.8c | 0.11c | 0.28d | 0.23d | 0.18d | 0.37d | 1.1c |  |
|  | 288 | 18.0e | 33.0f | 10f | 25.5e | 1.7d | 5.7f | 0.86e | 2.3e | 0.33f | 2.2d | 0.07e | 0.21e | 0.18e | 0.17d | 0.24e | 0.93e |  |
| **LSD** | | 3.45 | 2.3 | 2.78 | 24.1 | 0.56 | 1.23 | 0.31 | 1.41 | 0.76 | 1.2 | 0.88 | 0.3 | 4.11 | 0.9 | 1.23 | 0.14 |  |
| **F value** | |  |  |  |  |  |  |  |  |  |  |  |  |  |  |  |  |  |
| Un inoculated (df=1) | | 156 | 76.4 | 182 | 221 | 72.1 | 21.3 | 76.4 | 76.2 | 35.2 | 15.6 | 145 | 654 | 118 | 53.2 | 56.2 | 542 |  |
| Inoculated (df=3) | | 78.2 | 143 | 113 | 71.2 | 19.0 | 52.1 | 56.7 | 32 | 234 | 122 | 341 | 76.9 | 23.1 | 82.1 | 113 | 211 |  |
| Un inoculated x inoculated (df=3) | | 7.32 | 178 | 23.2 | 13.2 | 7.3 | 87.6 | 24.5 | 14.2 | 564 | 5.11 | 112 | 65.4 | 18.2 | 34.1 | 45.6 | 55.1 |  |

In this and succeeding tables each value is a mean of three replicates where each replicate constituted three plants/pot. Values denoted with different letters are significantly (*p* ≤ 0.05) different according to Duncan’s multiple range test (DMRT).

**Supplementary Figures**

**Fig. S1:** Linear regression between biological parameters of chickpea plants grown in sandy clay loam soil treated with varying concentrations of fungicide and inoculated with *M. ciceri* BRM5;

1. nodule number vs root length (R^2^=0.85)
2. nodule number vs shoot length (R^2^=0.68)
3. nodule number vs nodule biomass (R^2^=0.91)
4. root biomass vs total chlorophyll content (R^2^=0.77)
5. root biomass vs carotenoid content (R^2^=0.93)
6. total dry biomass vs P content (R^2^=0.71)
7. total dry biomass vs N content (R^2^=0.87)
8. total dry biomass vs seed yield (R^2^=0.84)
9. seed yield vs grain protein (R^2^=0.81)

**Fig. S2:**  Loading (A) and score (B) plots showing the principal component analysis (PCA) of various parameters of *C. arietinum* plants under KITZ stress and bio inoculated with *M. ciceri* BRM5 strain. Here, SVI seedling vigor index, RL root length, SL shoot length, RFW root fresh weight, SFW shoot fresh weight, RDW root dry weight, SDW shoot dry weight, chl, total chlorophyll, corot carotenoid content, NDB nodule biomass, LHb leghaemoglobin content, SY seed yield, GP grain protein, RN, root nitrogen, SN shoot nitrogen, RP root phosphorous, SP shoot phosphorous, R pro, root proline, S pro shoot proline, L pro leaf proline, G pro grain proline, APX ascorbate peroxidase, GPX guaiacol peroxidase, POD peroxidase, CAT catalase and MDA malondialdehyde.

**Figure. S3:** Colonization of *M. ciceri* strain BRM5 on root surface of *C. arietinum* plants using scanning electron microscopy; uninoculated control showing no colonization on roots (**panel A**) inoculated and treated with kitazin showing adherence of bacteria on root surface (**panel** **B**).
